# The Dead Time Characterization Method of Quartz Flexure Accelerometers Using Monotonicity Number

**DOI:** 10.3390/s19143123

**Published:** 2019-07-15

**Authors:** Bin Wu, Lingyun Ye, Tiantian Huang, Zhaowei Yang, Kaichen Song

**Affiliations:** 1 College of Biomedical Engineering & Instrument Science, Zhejiang University, Hangzhou 310027, China; 2 Science and Technology on Special System Simulation Laboratory, Beijing Simulation Center, Beijing 100854, China

**Keywords:** quartz flexure accelerometer, system identification, dead time

## Abstract

Dead time estimation is important in the design process of quartz flexure accelerometers. However, to the authors’ knowledge, the dead time existing in quartz flexure accelerometers is not well investigated in conventional identification studies. In this paper, the dead time, together with the open-loop transfer function of quartz flexure accelerometers, is identified from step excitation experiments using two steps. Firstly, a monotonicity number was proposed to estimate the dead time. Analysis showed that the monotonicity number was robust enough to measurement noise and sensitive to step excitation. Secondly, parameters of the open-loop transfer function were identified using the least mean squares algorithm. A simulation example was applied to demonstrate the validity of the proposed method. The verified method was used to test a quartz flexure accelerometer. The experimental result shows that the dead time was 500 μs.

## 1. Introduction

Since their development in the 1990s, quartz flexure accelerometers (QFAs) have been widely used in many fields, such as inertial navigation systems [1], the drilling industry [2,3], and microgravity measurements [4,5,6]. Conventionally, QFAs output a current signal proportional to the input acceleration. An analog-to-digital converter is required between the accelerometer and the digital signal processor. This analog-to-digital converter is not included in the closed-loop of QFA, hence, the drift of the converter directly degrades the accuracy of the acceleration measurements. To overcome this drawback, a structure that involves the converter in a closed-loop QFA has been proposed and developed in recent years [7,8]. Compared to conventional QFAs, the new structure digitalizes the differential capacitance signal and uses a digital signal to drive the rebalance force, so a more advanced control algorithm is able to be applied. Parameters of the control algorithm are able to be adjusted according to the transfer function of a specific accelerometer. There are two ways to establish the transfer function. One way is to use the differential equations and collect all the physical parameters required. This method is not feasible in practice, because the mechanism part of the QFA is enclosed in an airtight shell. The other way is to identify the transfer function based on experimental data. Many studies have been conducted on identification methods for QFAs [9,10,11]. However, these studies have neglected dead time in the identifying process. As is well known, dead time has a significant bearing on the achievable performance for control systems [12,13,14]. Hence, this paper develops a method of estimating the dead time and the transfer function of QFAs.

System identification requires an excitation signal and an identification algorithm. There are various kinds of excitation signals used in the system identification process, such as pulse, sinusoidal, pseudo-random binary sequence, and step [15]. Compared with other excitation signals, step signal is more widely used for two reasons: first, step signal is a sufficient excitation signal [16]; second, step signal is simple for realization in embedded systems. For example, Rake [17] describes a graphical identification method based on step response. Parameters and dead time are calculated from response curvature. It is simple but sensitive to measurement noise. To solve this problem, Bi et al. [18] developed a robust identification method for a first-order plus dead time model from step response. It is a method derived from an integral equation approach, and it inherits the characteristic of being robust to measurement noise. However, only a first-order model was considered in this study. Similar work was done by Wang et al. [19]. The parameters and dead time were obtained from a set of linear regression equations, and a second-order plus dead time model was studied as an example. All the works mentioned above focused on a continuous system, while a discrete system is more common in fields like embedded control systems. For a discrete system, the identification process is more direct, since the regression matrix simply contains shifted samples of the stored input and output data [20]. Elnaggar et al. [21] proposed a recursive method to estimate the discrete dead time and parameters. This recursive method assumes an initial dead time value and identifies the parameters using least mean squares (LMS), then minimizes the prediction error with respect to the dead time value. The main drawback is that iterations of the dead time value increase the computation cost [22]. Besides, measurement noise has a significant effect on estimation accuracy in this method. A similar method was applied to a thermodynamic process [23]. 

In this paper, step signal was used as the excitation signal. Dead time estimation and parameter identification were completed in discrete time domain. The model to be identified was a second-order plus dead time model [24]. A method based on monotonicity number was proposed to estimate the dead time. The concept of monotonicity number is studied in the field of mathematics [25], but has not found its application in the field of signal processing. Analysis shows that the position of the first decrease in a sequence of continuous numbers generated by a random variable is the Euler number e, independent of the distribution of the random variable. Consequently, the dead time estimation method developed from monotonicity number is inherently robust to measurement noise. Furthermore, it does not require iterations, which helps reduce computation load compared to the recursive method. After the dead time is estimated, parameters are identified using the LMS method. The accuracy of the parameter identification results is improved due to the fact that the dead time is removed from the original response data. These high-quality estimation and identification results provide essential information for dead time compensation algorithms, such as the Smith predictor [26,27], and promise the performance of digital control algorithms applied in QFAs.

The rest of this paper is organized as follows: Section 2 describes the diagram of a QFA and analyzes the transfer function of each part individually. Section 3 presents the dead time estimation method and the system identification algorithm. Section 4 studies the validity of the proposed method and algorithm through simulations. Section 5 develops a prototype circuit and gives the experimental results. Section 6 presents related discussions. Finally, the main conclusions are drawn in Section 7.

## 2. System Description of Quartz Flexure Accelerometer Based on New Structure

**Figure 1:**
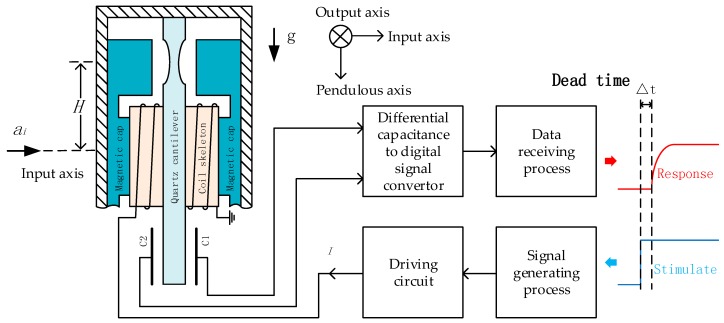
Diagram of the dead time test system.

The diagram of a QFA based on the new structure is shown in Figure 1. It is composed of two parts: a mechanical structure and a servo circuit. The core of the mechanical structure is a pendulum which is very thin at the joint part (down to 0.02 mm at the state of the art). When the proof mass accelerates, an inertial force proportional to the acceleration causes a displacement at the end of the pendulum. This displacement leads to a change of differential capacitance, which is detected by the servo circuit. In closed-loop condition, an electromagnetic force driven by the differential capacitance signal is applied to the pendulum to keep the pendulum at the balance position. In this paper, the system works in an open-loop condition. The electromagnetic force is driven by a step signal that is generated by a digital signal processor, rather than the differential capacitance signal. The acceleration input axis is set to be perpendicular to the direction of gravity, hence, no inertial force is applied to the pendulum at the balance position. The step response is measured through differential capacitance to the digital signal convertor. By repeating the step experiments, the step responses are averaged to improve the measurement noise. Using the averaged step excitation signals and the step responses, the dead time is estimated and the open-loop transfer function is identified.

From the signal generating process to the data receiving process, the open-loop system consists of the following parts: digital-to-analog converter, driving circuit, torque, pendulum, differential capacitor, capacitance measuring circuit, and capacitance transform module. Among these parts, the digital-to-analog converter, driving circuit, capacitance measuring circuit, and capacitance transform module form the servo circuit. Parameters of the serving circuit can be measured conveniently. Torque and pendulum form the mechanism part. The parameters of the mechanical part vary from one accelerometer to another, requiring to be identified through experiments.

In this paper, the experiments were driven by digital signals, $D_{d}$. A step signal was adopted as the driving signal, and it was generated by a digital signal processor. The step signal starts from a level where bias of the driving circuit is eliminated. To limit the movement of the pendulum, amplitude of the step signal is set to be small because the pendulum is very sensitive in the open-loop condition. Hence, the signal-to-noise ratio is very low for the response data of a single experiment. By repeating the step experiments, the signal-to-noise ratio is improved, which helps raise the accuracy of the parameter identification results. 

The digital driving signal, $D_{d}$, is converted into a corresponding voltage, V, by a digital-to-analog converter. Digital output 1 corresponds to 2.5 V, while −1 corresponds to −2.5 V. Hence, gain of the digital-to-analog converter is given by:

(1) $$K_{v}=\frac{V}{D_{d}}=2.5.$$

**Figure 2:**
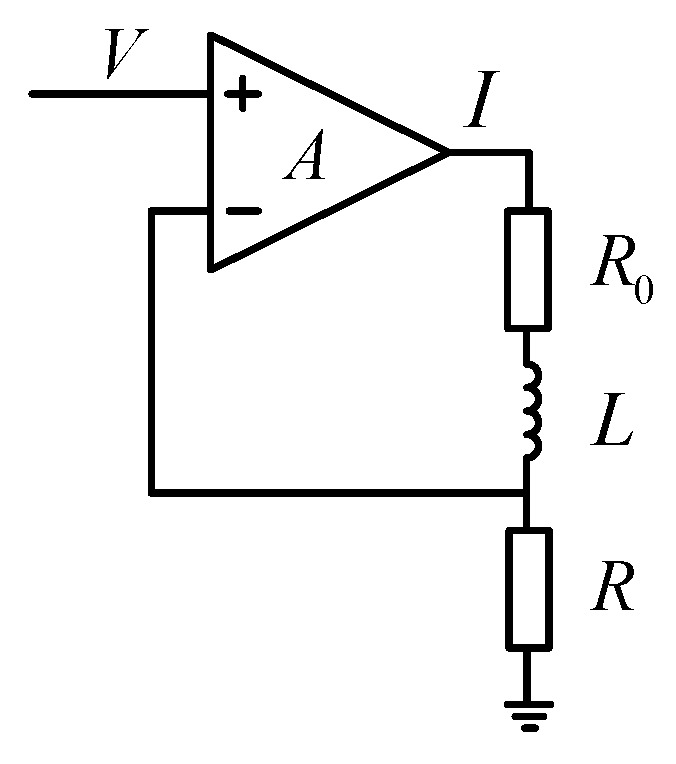
Schematic diagram of the driving circuit.

Following the digital-to-analog converter is a driving circuit, which transforms control voltage to torque current. Figure 2 shows the schematic diagram of the driving circuit. Let V be the input voltage and I be the output current. The transfer function of the driving function is expressed as:

(2) $$K_{i}\left(s\right)=\frac{I\left(s\right)}{V\left(s\right)}=\frac{A}{Ls+RA+R+R_{0}},$$

where resistor R is a sample resistor of 50 Ω. $R_{0}$ and L are coil resistance and inductance respectively, A is the open-loop gain of the amplifier. s is the Laplace operator. Since common amplifiers can easily achieve a gain bigger than 100 dB, the transfer function of the driving circuit is simplified as:

(3) $$K_{i}\left(s\right)=\frac{I\left(s\right)}{V\left(s\right)}\approx \frac{1}{R}.$$

Hence, the gain of the driving circuit is approximately a factor of 0.02.

The current generated by the driving circuit produces an electromagnetic force, F, which is used to balance the inertial force in closed-loop conditions. The relation of the electromagnetic force, F, and the inertial acceleration, $a_{i}$, in closed-loop conditions is given by:

(4) $$\frac{FH}{P}=\frac{IK_{t}H}{P}=-a_{i},$$

where H is the length from hinge point to the center of gravity of proof mass m. F is the inertial force F along the input axis. Inertial force F is equivalent to the product of proof mass m and acceleration $a_{i}$. Pendulosity P is equivalent to the product of proof mass m and pendulum length H. At the balance position, 1 g acceleration corresponds to 1 mA driving current. Hence, the product of $K_{t}$ and $\frac{H}{P}$ is equivalent to 1000. Considering Equations (1) and (3), the ratio of $a_{t}$ to *D_d_* is 50.

Acceleration generated by the electromagnetic force, as well as the acceleration generated by the inertial force, causes the movement of the pendulum. The pendulum is the core of QFAs. It is a high-purity quartz disc connected to the rigid outer frame by two thin hinges. The dynamic equation of the pendulum is derived from Newton’s law of motion. As a result, angular θ is the solution of the following second-order differential equation:

(5) $$J\ddot{\theta }+C\dot{\theta }+K\theta =P\left(a_{i}-a_{t}\right),$$

where J is the moment of inertia. It is equivalent to the product of proof mass m and square of the pendulum length H. C is the damping coefficient. It determines the torque needed for an angular velocity about output axis. K is the rotational stiffness. It is the relation between an applied torque and the corresponding angular. These three parameters cannot be measured directly, and vary from product to product. These parameters need to be identified through experiments. In open-loop experiments, the inertial acceleration $a_{i}$ is zero, and the transfer function of the pendulum is expressed as:

(6) $$K_{\theta }\left(s\right)=\frac{\theta \left(s\right)}{a_{t}\left(s\right)}=P\frac{1}{Js^{2}+Cs+K}.$$

When the pendulum rotates at an angle θ near the balance position, the moving plate is assumed to be parallel with other fixed plates [28,29]. Consequently, the differential capacitance is expressed as:

(7) $$\Delta C=C_{1}-C_{2}=\frac{\varepsilon_{0}\varepsilon_{r}S}{d-\Delta d}-\frac{\varepsilon_{0}\varepsilon_{r}S}{d+\Delta d}\approx 2C_{0}\frac{\Delta d}{d},$$

where $\varepsilon_{0}$ and $\varepsilon_{r}$ are vacuum permittivity and relative permittivity, respectively, S is the area of plates, d is the distance between the moving plate and the fixed plate, Δd is the movement of the pendulum, $C_{0}$ is the initial capacitance of $C_{1}$ and $C_{2}$. As Δd≈θz, the ratio of the change of differential capacitance ΔC to the angle θ is expressed as:

(8) $$K_{c}=\frac{\Delta C}{\theta }\approx 2C_{0}\frac{z}{d},$$

where z is the distance between the joint point of the pendulum and the center of plates.

The differential capacitance is measured by a capacitance-to-digital converter. The converter gives the ratio of $C_{1}$ to $C_{2}$. In the digital signal processor, the measurement $D_{r}$ is processed by:

(9) $$D_{c}=\frac{1-D_{r}}{1+D_{r}},$$

which leads to the relation of differential ΔC and measurement $D_{c}$:

(10) $$D_{c}=\frac{\Delta C}{C_{0}}.$$

Considering Equation (8), the relation of angle θ and measurement $D_{c}$ is expressed as:

(11) $$D_{c}=2\frac{z}{d}\theta .$$

Let $K_{a}=100\frac{z}{d}P$, the open-loop transfer function $K_{o}\left(s\right)$ is given by:

(12) $$K_{o}\left(s\right)=\frac{K_{a}}{Js^{2}+Cs+K}.$$

**Figure 3:**
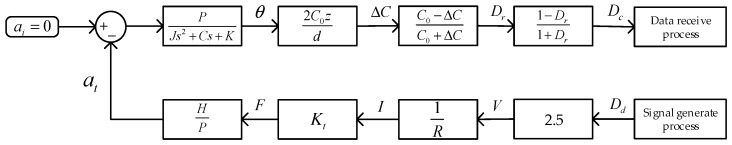
Diagram of the open-loop transfer function.

Corresponding to Equation (12), the block diagram of the open-loop transfer function is shown in Figure 3.

Adding on the dead time existing in the forward path of the open-loop, transfer function $K_{d}\left(s\right)$ is expressed as:

(13) $$K_{d}\left(s\right)=e^{-\tau s}\frac{K_{a}}{Js^{2}+Cs+K}.$$

The dead time of quartz flexure accelerometer τ is defined as the delay from when step excitation command $D_{d}$ is issued until when digital measurement result $D_{c}$ first begins to respond. Using the Tustin method [30], the discrete model of the open-loop transfer function is expressed as:

(14) $$K_{d}\left(z\right)=z^{-\frac{\tau }{T}}\frac{K_{a}T^{2}{\left(1+z\right)}^{2}}{\left(4J+2CT+KT^{2}\right)z^{2}+\left(2KT^{2}-8J\right)z+\left(4J-2CT+KT^{2}\right)},$$

where T is the sampling interval. In this paper, the dead time was assumed to be an integer multiple of sampling interval, and the fractional part was neglected. The reason was that the usual z-transform theory cannot handle fraction powers.

## 3. Dead Time Estimation and Parameter Identification

### 3.1. Dead Time Estimation Using Monotonicity Number

To obtain the open-loop transfer function presented in Equation (14), the dead time is firstly estimated and the parameters are then identified. A monotonicity number (MN) is proposed to estimate the dead time existing in the forward path of the open-loop. For a series of discrete data, the k-th monotonicity number is defined as the number of data that keep monotonic following the current data with index k. There are two kinds of monotonicity numbers. The increasing monotonicity number (IMN) is defined as:

(15) $$IMN\left(k\right)=n_{max}-k,n_{max}=\max\{n\in N|n\geq k,\;\forall j\in \left[k,n\right],\;i\in \left[k,n\right],\;\frac{x_{j}-x_{i}}{j-i}\geq 0\}.$$

In contrast, the decreasing monotonicity number (DMN) is defined as:

(16) $$DMN\left(k\right)=n_{max}-k,n_{max}=\max\{n\in N|n\geq k,\;\forall j\in \left[k,n\right],\;i\in \left[k,n\right],\;\frac{x_{j}-x_{i}}{j-i}\leq 0\}.$$

For example, given a time series:

(17) X={2, 3, 1, 2, 3, 4, 5},

the increasing monotonicity number vector of X is:

(18) IMN={1, 0, 4, 3, 2, 1, 0}.

According to Equation (15), the whole sample space of IMN is given by:

(19) $$S_{IMN}:\left\{0,\;1,\;2,\;3,\;4,\;\cdots \right\}.$$

The probability of any sample is given by:

(20) $$P\left(IMN=n-k\right)=\int_{-\infty }^{\infty }f\left(x_{k}\right)\cdots \int_{x_{n-2}}^{\infty }f\left(x_{n-1}\right)\int_{x_{n-1}}^{\infty }f\left(x_{n}\right)\int_{-\infty }^{x_{n}}f\left(x_{n+1}\right)dx_{n+1}dx_{n}dx_{n-1}\cdots dx_{k},$$

where $x_{k}$ denotes the randomized trial of the current data in the time series, and $x_{k\;+\;1}$ denotes the randomized trial of the next data, etc. The function $f\left(x_{n}\right)$ denotes the probability density function (PDF) of $x_{n}$. Here, the measurement noise is assumed to obey normal distribution, which means:

(21) $$f\left(x_{n}\right)=\frac{1}{\sigma \sqrt{2\pi }}e^{-\frac{{\left(x_{n}-\mu \right)}^{2}}{2\sigma^{2}}}.$$

For normal distribution noise, the first seven probabilities of $S_{IMN}$ are calculated in Table 1. When the increasing monotonicity number increases, the corresponding probability decreases rapidly. It illustrates that the increasing monotonicity number of noise is mostly less than or equal to 6. An interesting fact is that these probabilities are not influenced by the standard deviation and the mean value.

When a step excitation occurs, the monotonicity number of output data rises right after the dead time. The amplitude of monotonicity number excited by step signal is much bigger than that of the noise because the open-loop system is very sensitive. Hence, the dead time is obtained by measuring the delay between the index where step excitation occurs and the index where monotonicity number rises suddenly.

### 3.2. Parameter Identification Based on the LMS Method

After the dead time is calculated, the discrete transfer function given in Equation (14) is identified by the least mean squares method. Let $\frac{\tau }{T}=q$, the Equation (14) is rewritten as:

(22) $$D_{c}\left(k\right)=\left(-D_{c}\left(k-1\right),\;-D_{c}\left(k-2\right),\;D_{d}\left(k-q\right)+2D_{d}\left(k-q-1\right)+D_{d}\left(k-q-2\right)\right){\left(a_{1},\;a_{2},\;b_{0}\right)}^{T}+w\left(k\right),$$

where $D_{c}$ and $D_{d}$ are measurement data and excitation signal, respectively, w(k) is the measurement noise. Parameters $a_{1}$, $a_{2},$ and $b_{0}$ are given by:

(23) $$a_{1}=\frac{2KT^{2}-8J}{4J+2CT+KT^{2}},$$

(24) $$a_{2}=\frac{4J-2CT+KT^{2}}{4J+2CT+KT^{2}},$$

(25) $$b_{0}=\frac{K_{a}T^{2}}{4J+2CT+KT^{2}},$$

Let L denote the length of experimental data, then measurement data and excitation data form equations:

(26) $$\left(\begin{array}{c} D_{c}\left(q+3\right)\\ D_{c}\left(q+4\right)\\ \cdots \\ D_{c}\left(L\right) \end{array})=(\begin{array}{ccc} -D_{c}\left(q+2\right), & -D_{c}\left(q+1\right) & D_{d}\left(3\right)+2D_{d}\left(2\right)+D_{d}\left(1\right)\\ -D_{c}\left(q+3\right), & -D_{c}\left(q+2\right) & D_{d}\left(4\right)+2D_{d}\left(3\right)+D_{d}\left(2\right)\\ \cdots , & \cdots , & \cdots ,\\ -D_{c}\left(L-1\right), & -D_{c}\left(L-2\right) & D_{d}\left(L-q\right)+2D_{d}\left(L-q-1\right)+D_{d}\left(L-q-2\right) \end{array})(\begin{array}{c} a_{1}\\ a_{2}\\ b_{0} \end{array})+(\begin{array}{c} w\left(q+3\right)\\ w\left(q+4\right)\\ \cdots \\ w\left(L\right) \end{array}\right)$$

In matrix form, let:

(27) $$y={\left(D_{c}\left(q+3\right),\;D_{c}\left(q+4\right),\;\cdots ,\;D_{c}\left(L\right)\right)}^{T},$$

(28) $$x={\left(a_{1},\;a_{2},\;b_{0}\right)}^{T},$$

(29) $$A=\left(\begin{array}{ccc} -D_{c}\left(q+2\right), & -D_{c}\left(q+1\right) & D_{d}\left(3\right)+2D_{d}\left(2\right)+D_{d}\left(1\right)\\ -D_{c}\left(q+3\right), & -D_{c}\left(q+2\right) & D_{d}\left(4\right)+2D_{d}\left(3\right)+D_{d}\left(2\right)\\ \cdots , & \cdots , & \cdots ,\\ -D_{c}\left(L-1\right), & -D_{c}\left(L-2\right) & D_{d}\left(L-q\right)+2D_{d}\left(L-q-1\right)+D_{d}\left(L-q-2\right) \end{array}\right),$$

(30) $$w={\left(w\left(q+3\right),\;w\left(q+4\right),\;\cdots ,\;w\left(L\right)\right)}^{T}.$$

Equation (26) is expressed as:

(31) y=Ax+w.

According to the least-mean-square principle, the optimal estimation is given by:

(32) $$\hat{x}={\left(A^{T}A\right)}^{-1}A^{T}y.$$

The covariance matrix of the estimation is expressed as:

(33) $$\Sigma_{x}={\left(A^{T}A\right)}^{-1}\Sigma_{y},$$

where $\Sigma_{y}$ is the covariance matrix of measurement noise. To improve the measurement noise, step excitation experiments are repeated and measurement data are averaged.

## 4. Simulation

### 4.1. Monotonicity Numbers of Normal Distribution Noises

**Figure 4:**
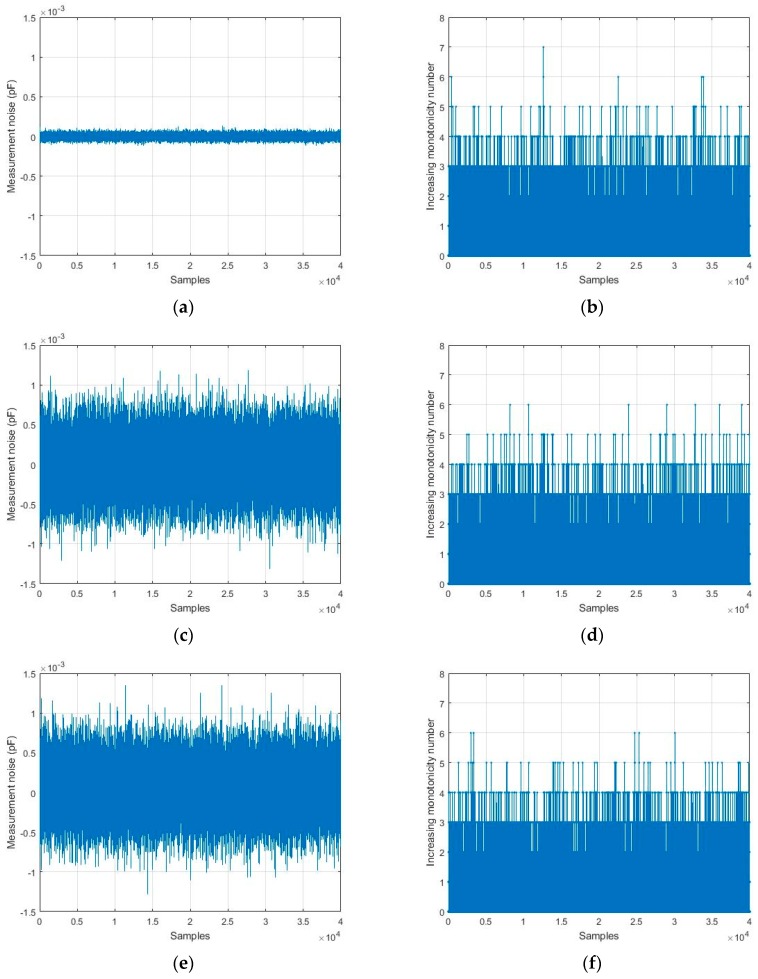
Three kinds of normal distribution noises and the corresponding increasing monotonicity numbers (IMN), (**a**) the noise that obeys $N\left(0,\;30^{2}\right)$ aF, (**b**) IMN of the noise that obeys $N\left(0,\;30^{2}\right)$ aF, (**c**) the noise that obeys $N\left(0,\;300^{2}\right)$ aF, (**d**) IMN of the noise that obeys $N\left(0,\;300^{2}\right)$ aF, (**e**) the noise that obeys $N\left(50,\;300^{2}\right)$ aF, (**f**) IMN of the noise that obeys $N\left(50,\;300^{2}\right)$ aF.

As illustrated in Table 1, the increasing monotonicity number of measurement noise is not influenced by its standard deviation and mean value. This conclusion is demonstrated by a simulation, which is shown in Figure 4. Three kinds of normal distribution noises are generated, and their increasing monotonicity number is calculated for comparison.

The simulation result shows that the monotonicity numbers are kept in the same and limited range, although the distributions of measurement noises are different. The monotonicity number is usually smaller than seven. Hence, it is reasonable to set eight as the threshold of response to step excitation when the measurement noise is Gaussian white noise. In practice, measurements may be corrupted by 1/f noise and drift noise. An alternative way of replacing the constant threshold is by judging from the ratio of the signal’s monotonicity number to the noise’s monotonicity number.

### 4.2. Dead Time and Parameter Identification Methods

A simulation w applied to verify the dead time estimation and parameter identification methods. The parameters used in the simulation are listed in Table 2. 

Using Equation (14), the discrete model under a 10 kHz sample frequency is given by:

(34) $$K_{d}\left(z\right)=z^{-6}\frac{0.0226+0.0452z^{-1}+0.0226z^{-2}}{1-1.785z^{-1}+0.785z^{-2}}.$$

This discrete model was excited by a step signal. The amplitude of the step signal was $5\times 10^{-5}$. This step signal drove the digital-to-analog converter to produce a stimulating force, which generated an acceleration of 2.5 mg. This stimulating acceleration was very small, because the discrete model represents an open-loop transfer function and it is validated in a small range otherwise non-linear effects have to be included. 

**Figure 5:**
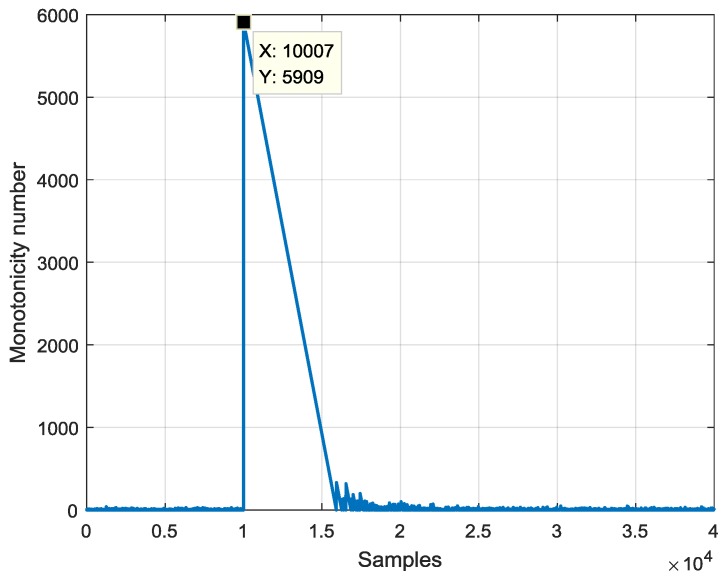
Simulation of dead time estimation with the noise that obeys $N\left(0,\;30^{2}\right)$ aF.

Firstly, given the simulation data, the dead time was estimated. Figure 5 shows the monotonicity number response to the step excitation. The step signal occurred at 1 s, which corresponded to the 10,000th sample. The monotonicity number rose at the 10,007th sample, and the amplitude at the 10,007th sample was much bigger than eight. Hence, the dead time estimation is seven samples with an error of one sample. 

The measurement data were corrupted by the measurement noise. The simulation results, which are shown in Table 3, illustrate that the dead time estimation method was robust to the measurement noise. When the measurement noise became bigger, the estimation error of Elnaggar’s method [21] became bigger, while that of the MN method proposed in this paper was not influenced.

Secondly, parameters of the discrete model were identified using the LMS method after the dead time was estimated. Using Equation (32), parameters $a_{1}$, $a_{2}$, and $b_{0}$ were fitted. The fitted transfer function is express as:

(35) $$K_{d}^{’}\left(z\right)=z^{-7}\frac{0.0227+0.0454z^{-1}+0.0227z^{-2}}{1-1.782z^{-1}+0.782z^{-2}}.$$

**Figure 6:**
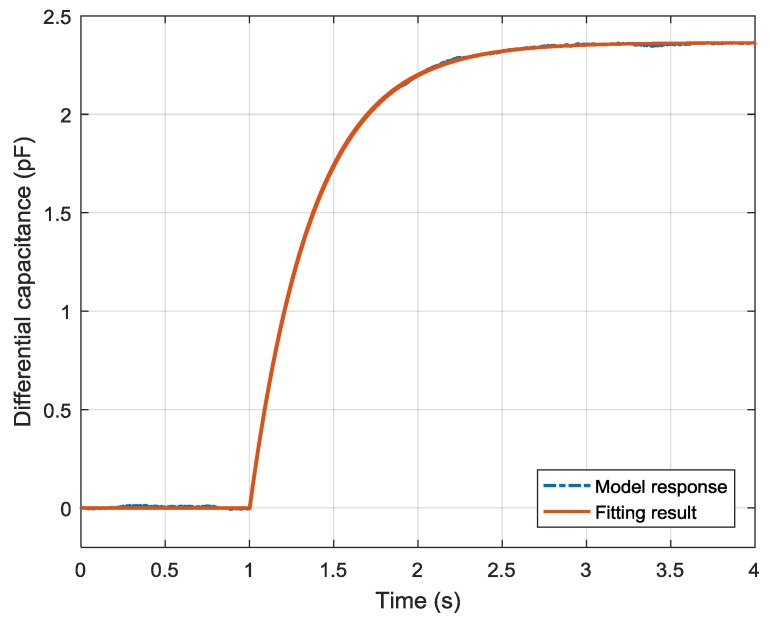
Comparison of model response and fitting result with noise that obeys $N\left(0,\;30^{2}\right)$ aF.

The fitting result is shown in Figure 6. Compared to the ideal model response, the root mean square error (RMSE) of the fitting result was 4.941 fF. With the fitting results of $a_{1}$, $a_{2},$ and $b_{0}$, the mechanism parameters J, C, K, and $K_{a}$ are given in Table 4.

In contrast to the dead time estimation method, the performance of the LMS method was affected by the measurement noise. More simulation results are listed in Table 5. When the measurement noise increased, the root mean square error increased proportionally. To improve the precision of the identification results, the step excitation experiment needs to be repeated and the response data need to be averaged.

## 5. Experimental Results and Discussions

**Figure 7:**
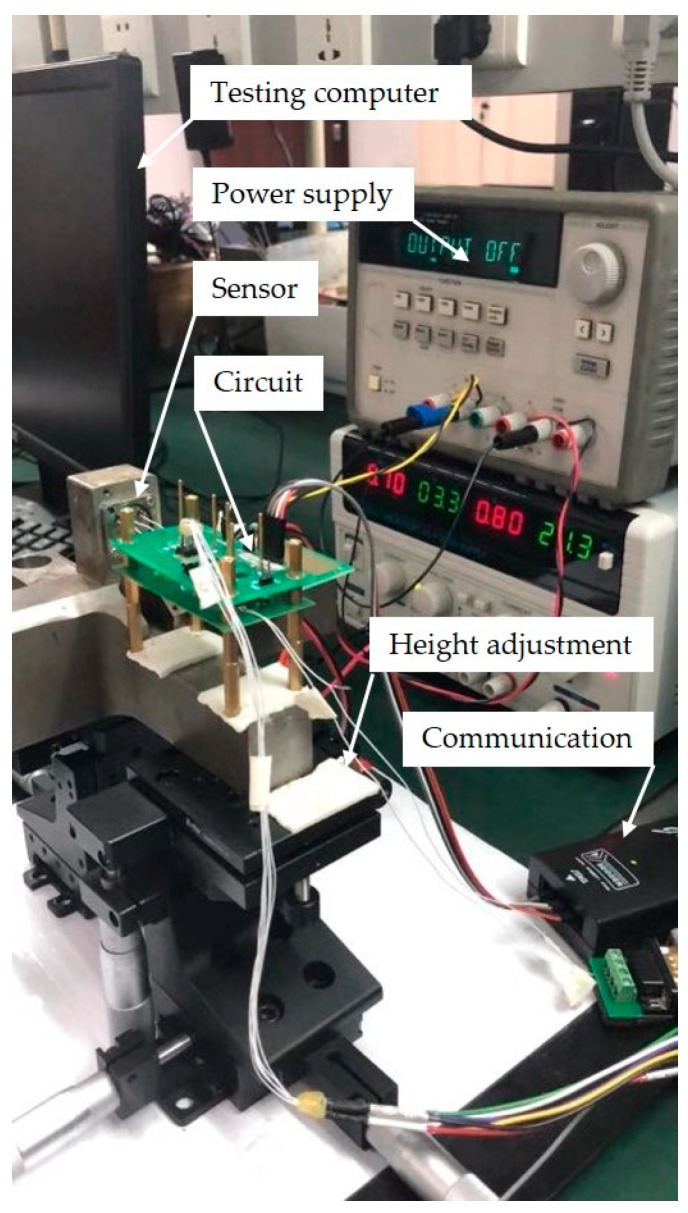
Experimental setup of the dead time test system.

A quartz flexure accelerometer was tested using the dead time estimation and parameter identification methods which were verified by simulation. The experimental setup is shown in Figure 7. The quartz flexure accelerometer was made up of two parts: the mechanism and the circuit. The mechanism part contains the quartz pendulum, coil skeleton, and magnetic cap, which are described in Figure 1. This sensor was mounted on an iron cross whose bottom was perpendicular to the mounting face. The iron cross was put on two height adjusting devices, hence, the bottom of the iron cross could be horizontal by adjusting the height. The circuit mainly performs two functions: driving the coil and measuring the capacitance. The coil was driven by the step signal repeatedly, and the capacitance was measured and transmitted to the data-processing computer. 

**Figure 8:**
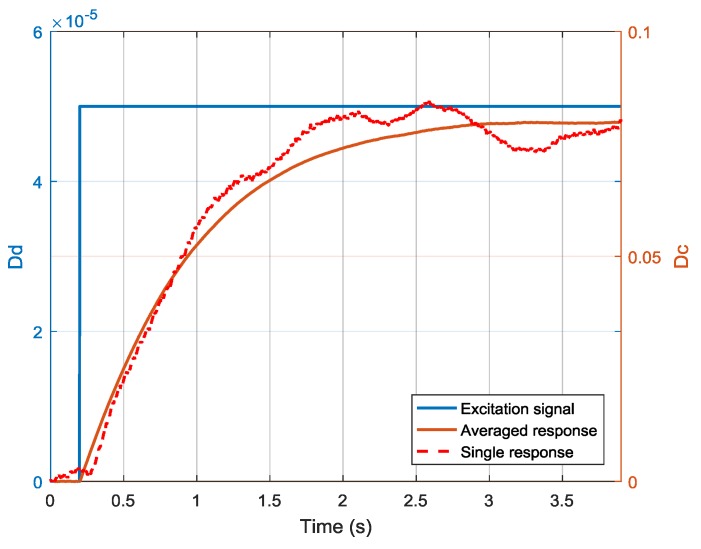
Excitation signal and responses of step experiments.

The experimental data are shown in Figure 8. Sample frequencies of the excitation signal $D_{d}$ and the response signal $D_{c}$ were both 10 kHz. Sample time was set to be 3.9 s. The amplitude of the step signal was $5\times 10^{-5}$, and it occurred at 0.2 s. The response was corrupted by measurement noise. To improve the dead time estimation and system identification results, 300 independent responses were averaged. The averaged response is plotted in a solid line while a single response is plotted in a dotted line.

**Figure 9:**
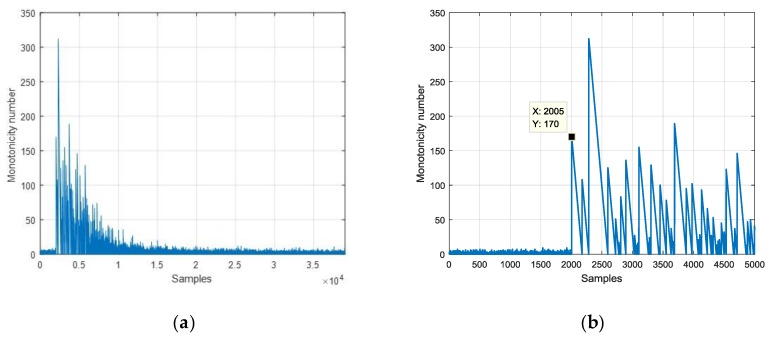
Monotonicity number of the averaged response data: (**a**) in the scale of 39,000 samples, (**b**) in the scale of 5000 samples.

Using the excitation data and the averaged response data, the monotonicity number of the experimental result is shown in Figure 9. The first peak of the monotonicity number appears at the 2005th sample. Since the step signal occurs at the 2000th sample, the dead time estimation is 5 samples.

Considering that the sample frequency was 10 kHz, the dead time existing in the open-loop was 500 μs. This dead time was composed of three parts: the dead time between step excitation command *D_d_* and actual step excitation current *I*, the dead time between actual step excitation current *I* and differential capacitance output ΔC, the dead time between differential capacitance output ΔC and digital measurement result *D_c_*. Among these three parts, the first part and the third part, which concern the electronic circuit, can be measured directly and individually. To measure the dead time in the first part, step excitation command *D_d_* was used to drive an extra I/O port of the digital signal processor, and the driving circuit was connected to a resistor of 150 Ω. The dead time from when the voltage of the I/O port sudden rises until when the voltage of the resistor first begins to respond was measured using an oscilloscope (Tektronix TBS 1102). Experimental result showed that the dead time of this part was 8 μs. To measure the dead time in the third part, a capacitor of 30 pF controlled by a switch was connected to the capacitance measuring circuit. When the switch was on, the circuit was connected to the capacitor; when the switch was off, the circuit was connected to the ground. The dead time of this part was obtained by measuring the delay between the switching signal and the sudden change of measurement *D_c_*. Experimental results showed that the dead time between differential capacitance output ΔC and digital measurement result *D_c_* was 20 μs. Consequently, the dead time between the actual step excitation current *I* and differential capacitance output ΔC, which was 472 μs in this experiment, made the most effective contribution to the whole dead time existing in the open-loop of the quartz flexure accelerometer.

**Figure 10:**
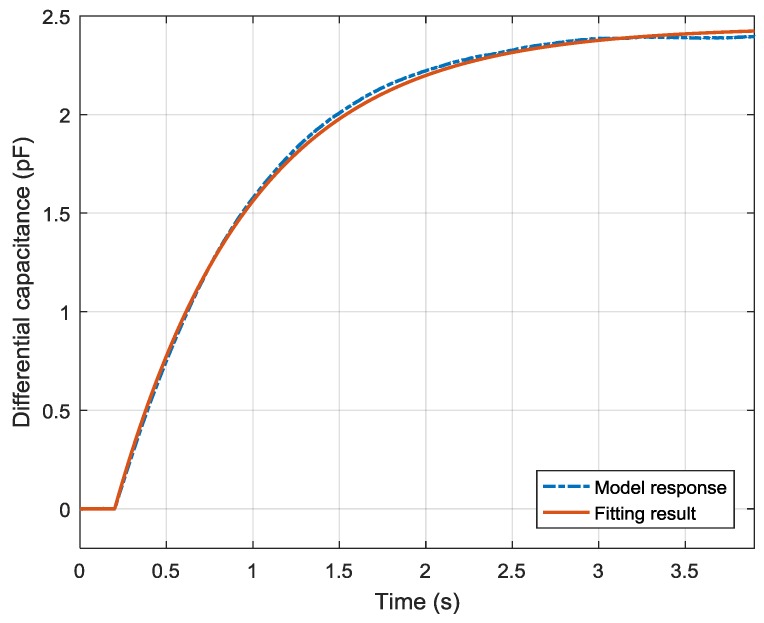
System identification result of the experimental data.

After the dead time was estimated, the system parameters were identified using the LMS method. The identification result is shown in Figure 10. The fitting result is plotted in the solid line while the experimental data is in the dashed line. Compared to the averaged response data, the RMSE of the fitting result was 18.906 fF. 

Combining the dead time estimation and parameter identification results, the discrete transfer function of DQFA (open-loop) is expressed as:

(36) $$K_{d}^{\prime \prime }\left(z\right)=z^{-5}\frac{0.0358+0.0716z^{-1}+0.0358z^{-2}}{1-1.310z^{-1}+0.310z^{-2}}.$$

With the fitting result of discrete transfer function, the mechanism parameters are given in Table 6. 

## 6. Discussion

The quartz flexure accelerometer based on the new structure achieved a significant development, because it overcame the precision loss inherently existing in the transforming process of analog quartz flexure accelerometers. However, the quartz flexure accelerometer based on the new structure demands more stringent modeling accuracy. Driven by this demand, researchers have developed methods identifying the mechanism parameters of QFAs, such as the moment of inertia, the damping coefficient, and the rotational stiffness. However, the dead time, which has a great effect on the control algorithm design, is not mentioned in previous studies. This paper puts effort in on the dead time estimation and parameter identification methods of QFAs to improve the modeling accuracy.

The dead time estimation method is mainly based on the monotonicity number, which means the number of data that keeps monotonic following the current data. Analysis shows that the monotonicity number was robust to measurement noise because the probability distribution of the monotonicity number was not influenced by the statistic characteristics of the measurement noise. Here, the measurement noise was assumed to be white noise (normal distribution noise). For other kinds of noise, the robustness of the monotonicity number is still unclear, and it is worthy of future study. Analysis also showed that the monotonicity number was sensitive to step excitation. In simulation, the monotonicity number of the excitation signal reached 5909, while that of measurement noise was kept in a limited range of eight. One drawback to this dead time estimation method is that it is only applicable to a discrete system. The fractional part of dead time was not considered. A solution to this problem is increasing the sample frequency, then the fractional part of dead time becomes smaller. Another solution presented in Reference [31] is increasing the order of the numerator by one and calculating the equivalent model.

The parameter identification method was mainly based on the least mean squares principle. In contrast to the dead time estimation method, the parameter identification method was affected by the measurement noise. Analysis showed that the root mean square error of the fitting result was proportional to the measurement noise. To overcome this problem, the step excitation experiment was repeated and the step response data were averaged. This solution was direct and effective, but not efficient. A more robust and efficient identification method, such as artificial neural networks [32,33], will be used in future work.

A simulation example was presented to confirm the validity of the methods proposed in this paper. Using the verified methods, a quartz flexure accelerometer was tested. The experimental result showed that the dead time was 500 μs. This time may vary from one accelerometer to another, but it should not be neglected. A compensation algorithm, such as the Smith predictor, requires prior knowledge of the dead time to compensate. Hence, an accurate dead time estimation method is important to the designing process of these algorithms. The experimental result also gives the open-loop transfer function. Derived from the transfer function, the moment of inertia was 1.9 × 10^−8^ N·m·s/rad, the damping coefficient was 2.0 × 10^−4^ N·m·s/rad, and the rotational stiffness was 3.0 × 10^−4^ N·m·s/rad. With these identified parameters, it was able to design the servo control algorithm regrading to the specific accelerometer.

## 7. Conclusions

This paper presented a study on the system identification of the quartz flexure accelerometer, especially considering the dead time which was not focused on in conventional studies. The dead time and mechanism parameters of the digital quartz flexure accelerometer were obtained through step excitation experiments. A monotonicity number, which was robust enough to measurement noise but sensitive to step excitation, was proposed to estimate the dead time. The mechanism parameters were identified using the least mean squares method. The validity of the dead time estimation and parameter identification methods was verified by simulation. A quartz flexure accelerometer was tested using the verified methods.

## Figures and Tables

**Table 1:** First seven probabilities of the sample space of the increasing monotonicity number.

The Increasing Monotonicity Number	Probability
0	1/2
1	1/3
2	1/8
3	1/30
4	1/144
5	1/840
6	1/5760

**Table 2:** Mechanism parameters used in the simulation.

$J\;(N\cdot m\cdot s^{2}/\mathrm{rad})$	C (N·m·s/rad)	K (N·m/rad)	$\mathrm{Ka}\;(N\cdot s^{2})$	τ (s)
$5.73\times 10^{-8}$	$1.38\times 10^{-4}$	$3.68\times 10^{-4}$	0.58	$6\times 10^{-4}$

**Table 3:** Effect of measurement noise on dead time estimation.

σ (aF)	Monotonicity Number Method	Elnaggar’s Method	Error of Monotonicity Number Method	Error of Elnaggar’s Method
3	7	6	1	0
30	7	7	1	1
300	7	16	1	10

**Table 4:** Identified mechanism parameters used in the simulation.

$J\;(N\cdot m\cdot s^{2}/\mathrm{rad})$	C (N·m·s/rad)	K (N·m/rad)	$\mathrm{Ka}\;(N\cdot s^{2})$	τ (s)
$5.56\times 10^{-8}$	$1.36\times 10^{-4}$	$3.61\times 10^{-4}$	0.57	$7\times 10^{-4}$

**Table 5:** Root mean square errors of fitting results under different measurement noise.

σ (aF)	Root Mean Square Error (fF)
3	0.489
30	4.941
300	49.437

**Table 6:** Identified mechanism parameters of the tested accelerometer.

$J\;(N\cdot m\cdot s^{2}/\mathrm{rad})$	C (N·m·s/rad)	K (N·m/rad)	$\mathrm{Ka}\;(N\cdot s^{2})$	τ (s)
$1.9\times 10^{-8}$	$2.0\times 10^{-4}$	$3.0\times 10^{-4}$	0.41	$5.0\times 10^{-4}$
